# SEOM guidelines on hydroelectrolytic disorders

**DOI:** 10.1007/s12094-014-1234-2

**Published:** 2014-10-11

**Authors:** R. De las Peñas, Y. Escobar, F. Henao, A. Blasco, C. A. Rodríguez

**Affiliations:** 1 Consorcio Hospitalario Provincial de Castellón, Castellón, Spain; 2 Hospital General Universitario Gregorio Marañón, Madrid, Spain; 3 Hospital Universitario Virgen de la Macarena, Sevilla, Spain; 4 Hospital General Universitario de Valencia, Valencia, Spain; 5 Hospital Universitario de Salamanca-IBSAL, Salamanca, Spain

**Keywords:** Hydroelectrolytic disorders, Hyponatremia, SIADH, Hypercalcemia

## Abstract

Hydroelectrolytic disorders are one of the most common metabolic complications in cancer patients. Although often metabolic alterations affecting various ions are part of the manifestations of the oncological disease, even in the form of paraneoplastic syndrome, we must not forget that very often, these disorders could be caused by various drugs, including some of the antineoplastic agents most frequently used, such as platin derivatives or some biologics. These guidelines review major management of diagnosis, evaluation and treatment of the most common alterations of sodium, calcium, magnesium and potassium in cancer patients. Aside from life-sustaining treatments, we have reviewed the role of specific drug treatments aimed at correcting some of these disorders, such as intravenous bisphosphonates for hypercalcemia or V2 receptor antagonists in the management of syndrome of inappropriate antidiuretic hormone secretion-related hyponatremia.

## Introduction

Hydroelectrolytic disorders are one of the most common metabolic complications in cancer patients. Although often metabolic alterations affecting various ions are part of the manifestations of the oncological disease, even in the form of paraneoplastic syndrome, we must not forget that very often, these disorders could be caused by various drugs, including some of the antineoplastic agents most frequently used, such as platin derivatives or some biologics. The frequently nonspecific clinical manifestations require careful diagnosis and evaluation. With regard to treatment, apart from support measures used to correct hydroelectrolytic alterations, it is now of special interest the appearance of drugs that can specifically correct such alterations including bisphosphonates, more powerful in the case of hypercalcemia, and more recently V2 receptor antagonists to correct syndrome of inappropriate antidiuretic hormone secretion (SIADH) hyponatremia. Below is a brief review of the main alterations of sodium, calcium, magnesium and potassium in cancer patients, with recommendations for each one based on the grade of evidence.

### Hyponatremia

#### Definition

Hyponatremia is defined as a serum sodium concentration below 135 mmol/l (mM). From a biochemical point of view, recent consensus considers hyponatremia as “mild” if values are between 130 and 135 mmol/l; “moderate” between 120 and 129 mmol/l and “severe” if below 120 mmol/l. As for the symptoms of hyponatremia, the condition could be classified as mild, moderate or severe according to their intensity [1]. Regarding the time for disease development, hyponatremia is considered “acute” if developed in less than 48 h or “chronic” if developed over a longer time (evidence level IV, recommendation grade D).

#### Etiology and pathophysiology

The primary pathophysiological mechanism of hyponatremia is a fluid balance disorder with a relative excess of body water compared to the total sodium content. Normally, it is caused by a vasopressin dysfunction—antidiuretic hormone or ADH—the activity of which is required for developing hyponatremia. There are three types of hyponatremia according to serum osmolarity: iso-osmolar hyponatremia, hyperosmolar isonatremia and hypotonic or hypo-osmolar hyponatremia. Hypotonic hyponatremia is most frequently encountered in clinical practice, especially in cancer patients. The most frequent cause of hypotonic hyponatremia in oncological patients is a SIADH, a paraneoplastic syndrome that can occur in many tumor types, mainly in lung cancer cases. There is a consensus on pathophysiological differentiation of hyponatremia, first of all based on serum osmolarity, prior to preparing a treatment plan, which is why initial diagnosis should start with the correct characterization of hyponatremia (evidence level IV, recommendation grade D).

#### SIADH definition

The definition of SIADH should meet the following criteria: (a) hypotonic hyponatremia with clinical euvolemia; clinical criteria for euvolemia are the absence of signs of hypovolemia (normal eye pressure values, normal venous pressure, no orthostatism) and no signs of hypervolemia either (ascites, edemas); (b) no diuretic intake; (c) no hypothyroidism, no adrenal insufficiency (hypocortisolism) or renal failure; (d) absence of physiological stimuli for ADH secretion (recent surgery, severe pain, ADH secretion stimulating drugs, etc.); (e) consistent biochemical data: serum Na <135 mmol/l; plasma osmolarity <275 mOsm/kg; urine osmolarity >100 mOsm/kg; urinary Na >40 mmol/l (in the presence of dietary sodium). Various expert committees have prepared consensus documents establishing that the diagnosis of SIADH in cancer patients should include each and every one of the above-mentioned items [1–5]. In the case of a clinical suspicion of SIADH, ion concentration and osmolarity should be determined in plasma and urine (evidence level IV, recommendation grade D).

#### Therapeutic management of SIADH in oncological patients

##### Acute and/or severe hyponatremia

Acute and/or severe hyponatremia is hyponatremia with mild/severe symptoms and/or hyponatremia <48 h. (SNa <120 mmol/l).

Our therapeutic choice should be based on the clinical condition, independently of the time to develop hyponatremia (acute/chronic) or of the severity confirmed by laboratory tests (mild/moderate/severe), although usually there is a correlation.

In the case of clinically severe hyponatremia or if SNa is below 120 mmol/l, it is recommended to initiate treatment at the hospital with an IV of 3 % normal saline hypertonic solution (SHS).There are no randomized or control trials confirming the efficacy of this method, but at least five series of cases and expert recommendations consider this choice as a first-line treatment under these circumstances [1–5]. It is recommended not to exceed sodium correction levels during the whole treatment duration, especially with hypertonic saline solution (HSS): the maximum plasma sodium augmentation level indicated is 10 mmol/l in 24 h and 18 mmol/l in 48 h (evidence level IV, recommendation grade D) [1–6].

During corrective treatment with HSS, the use of any other hyponatremia-specific treatment should be avoided, with the exception of furosemide which will be used in case of overload and heart failure (Evidence level IV, Recommendation Grade D).

##### Mild/moderate hyponatremia

Mild/moderate hyponatremia includes mild/moderate symptoms and/or hyponatremia >48 h (SNa >120 mmol/l).

The treatment goal is to normalize sodium values and improve symptoms in a situation that is not life threatening. Several treatment options are considered and, in any case, the recommended correction levels should always be taken into account.

Water restriction (WR) consists of the administration of a maximum amount of fluids of 800 ml/day. It is recommended by expert consensus [2] as an initial measure, even though there are no confirming studies (evidence level IV, recommendation grade D). Nevertheless, this is not incompatible with certain cancer treatments of tumors causing SIADH that require proper hydration. This is why there are expert groups that do not recommend the use of WR in SIADH secondary to chemosensitive neoplasias receiving specific treatments (evidence level IV, recommendation grade D).

Selective vasopressin V2 receptor antagonists, in particular tolvaptan, are recommended in patients who are not candidates for WR and whose clinical situation might last for a few days, more specifically cancer-associated chronic SIADH cases. This approval stems from the publication of results from two randomized placebo control clinical trials, in which its efficacy was demonstrated in the case of hyponatremia (SALT 1 and 2 studies). In subsequent analyses, the efficacy was confirmed in the case of patients with hyponatremia secondary to SIADH [6, 7]. There are two systematic reviews of studies that compare V2 antagonists with placebo (a total of 15 studies were included in both reviews, out of which only the SALT study was a control one) and five comparative studies published later, demonstrating a globally positive effect in favor of the V2 antagonist regarding the elevation of serum sodium levels with no impact on survival and with not a single case of osmotic pontine demyelination. The reviews outline the heterogeneity of the studies as well as possible population and treatment biases (evidence level 1b, recommendation grade B).

The use of other pharmaceutical drugs recommended for the treatment of hyponatremia is more controversial in the SIADH scenario: there are no meta-analyses or systematic reviews to assess the efficacy of lithium salts, urea, demeclocycline or loop diuretics. Their possible recommendation by consensus guidelines is based exclusively on acase series published [2] (evidence level IV, recommendation grade D).

**Fig. 1:**
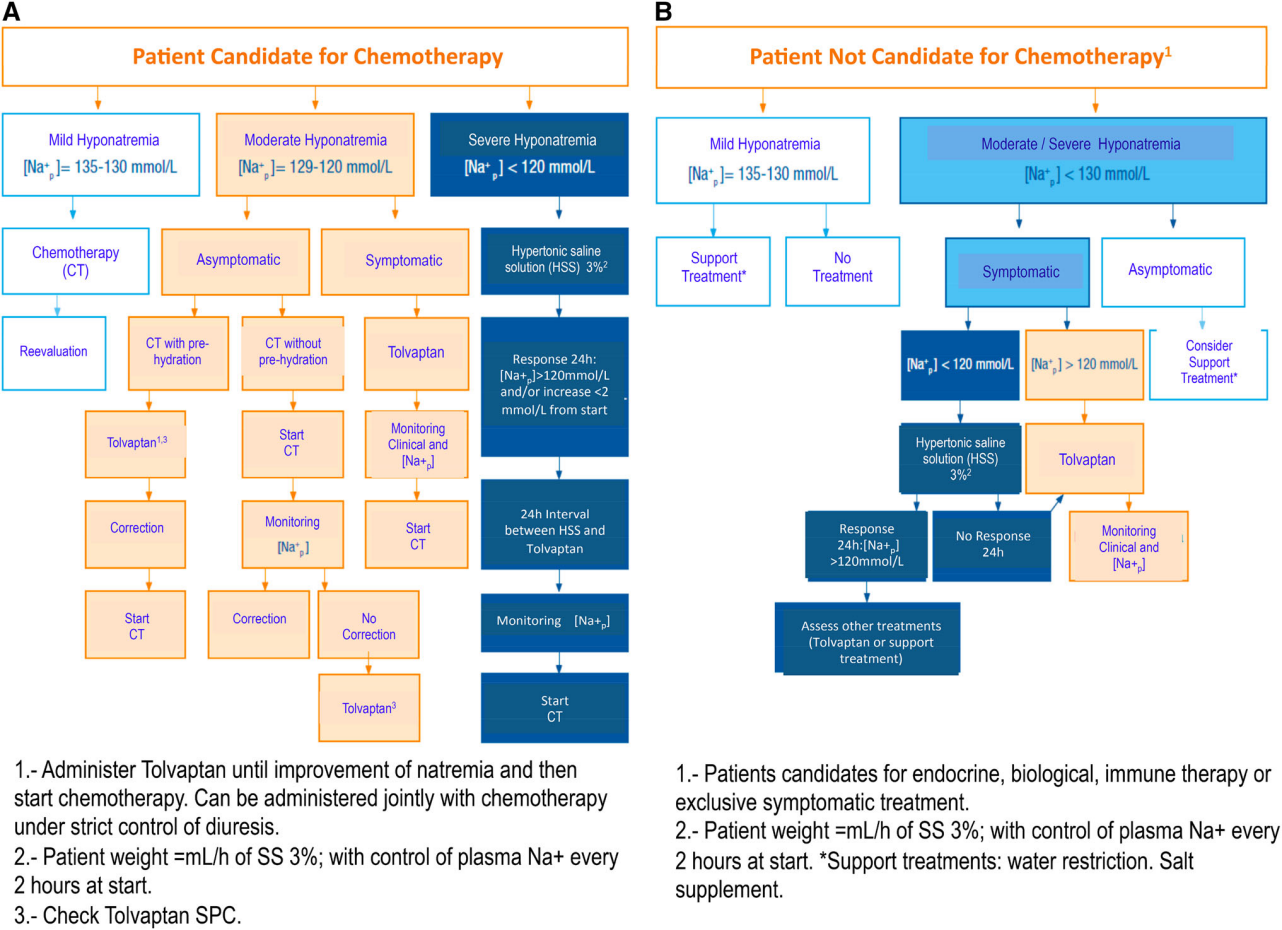
Treatment algorithm for hyponatremia in cancer patients. Taken from 1.

Recently, Spanish oncologists have published an algorithm for the treatment of hyponatremia, specifically designed for cancer patients. See Fig. 1a,b.

## Calcium disorders

### Hypercalcemia

Hypercalcemia is a common disorder in patients with cancer, occurring in approximately 20–30 % of cases, especially in the inpatient setting. The most common cancers associated with hypercalcemia are breast and lung cancer and multiple myeloma. Patients with hypercalcemia of malignancy have a poor prognosis.

#### Etiology, pathophysiology and clinical manifestations

Hypercalcemia can occur by three mechanisms: osteolytic metastases with local release of cytokines (including osteoclast activating factors); tumor secretion of parathyroid hormone-related protein (PTHrP) and tumor production of 1,25-dihydroxyvitamin D (calcitriol) [8]. Calcium in serum is bound to proteins. As a result, total serum calcium concentrations in patients with changes in serum albumin levels may not accurately reflect the physiological calcium concentration. The normal values are between 8.5 and 10.5 mg/dl (2.2–2.6 mmol/l).

Hypercalcemia may be associated with a broad spectrum of clinical manifestations, from few or no symptoms in patients with mild chronic hypercalcemia to severe disorders and coma. The degree of hypercalcemia, along with the rate of rise of serum calcium concentration, often determines symptoms and the urgency of therapy [9].

#### Treatment

The main initial measures to treat hypercalcemia include saline hydration and administration of calcitonin and a bisphosphonate. Sometimes, the use of glucocorticoids could be useful [10].

Patients with asymptomatic hypercalcemia (calcium <12 mg/dl) do not require immediate treatment. Patients may be advised about factors that can aggravate hypercalcemia (level of evidence IV, grade of recommendation D).

Asymptomatic or mildly symptomatic patients with calcium levels between 12 and 14 mg/dl require saline hydration, at an initial rate of 200 ml/h with urine control and prednisone can be added at a dose of 40 mg/day. Sometimes, loop diuretics may be used when patients have edema. Bisphosphonates are considered only if patients have bone metastases [11] (level of evidence IV. grade of recommendation D).

Patients with symptomatic hypercalcemia and/or calcium level greater than 14 mg/dl require more aggressive therapy. Volume expansion with isotonic saline to maintain urine output at 100 ml/h and 4 mg of zoledronic acid over 15 min is the most effective treatment [12]. (level of evidence IV, grade of recommendation D). Loop diuretics are recommended only if there is renal failure or heart failure. Calcitonin (4 UI/kg) could be useful if there is no change in calcium levels after the first 24–48 h. Dialysis is reserved for severe hypercalcemia with calcium levels greater than 18 mg/dl.

### Hypocalcemia

#### Etiology and clinical manifestations

Although there are multiple etiologies of hypocalcemia in oncological patients, one of the most frequent situations is hypocalcemia secondary to hypomagnesemia produced by the administration of treatments such as cisplatin or cetuximab. Clinical manifestations depend on the degree of hypocalcemia and the speed with which it is established. Chronic hypocalcemia is usually asymptomatic until a trigger occurs. Irritability, memory disorders, neurosis, psychosis, abnormal electrocardiogram (ECG) and tetany can be the most common signs and symptoms of hypocalcemia [13].

#### Treatment

Treatment must aim at correcting serum calcium and relieve patient symptoms. For mild symptomatic hypocalcemia, increasing oral calcium intake (250–500 mg/6 h) is recommended. For moderate and severe symptomatic hypocalcemia, the most recommended measures in an emergency are administration of 1–2 g of calcium gluconate 10 % over 10–20 min and then maintenance with 1 mg/kg/h. Concurrent hypomagnesemia must be treated at the same time.

## Magnesium disorders

Magnesium (Mg) is the fourth most abundant cation in the human body and the second one after potassium in the intracellular compartment [14]. It is essential for many enzymatic and metabolic processes. Mg concentration in plasma is in a narrow range between 1.7 and 2 mg/dl (0.7–1.4 mmol/l or 1.5–1.9 mEq/l) and homeostasis depends on the balance between intestinal absorption and renal excretion.

### Hypomagnesemia

#### Definition

Hypomagnesemia is defined as Mg plasma concentration below 1.7 mg/dl (<0.75 mmol/l or <1.5 mEq/l). Mild–moderate hypomagnesemia values range between 1.6 and 1 mg/dl and severe below 1 mg/dl.

#### Etiology and pathophysiology

Mg deficiency can be caused by three pathophysiological mechanisms [15]: decreased intake (dietary factor); diminished absorption due to resection of small intestine, cholestasis, pancreatic failure, diarrhea, stoma, fistula and others; and increased excretion such as in the case of alcoholism, diabetes mellitus, interstitial nephritis, polyuric phase of acute tubular necrosis, hyperthyroidism and hyperparathyroidism. This mechanism also occurs when nephrotoxic drugs are used such as aminoglycoside antibiotics, amphotericin, cyclosporine, loop diuretics and in cancer patients treated with some antitumor drugs, in particular cisplatin (CDDP) and anti-EGFR monoclonal antibodies (mAb).

Hypomagnesemia due to CDDP is related to the cumulative dose [16] and it is considered that the drug interferes with renal absorption of Mg through a lesion of the distal convoluted tubule (DCT), although some studies suggest that hypomagnesemia associated to CDDP could be the result of the gastrointestinal loss of Mg and not a renal loss [17].

Hypomagnesemia is also an adverse effect of cancer treatment with biologics, in particular anti-EGFR mAb (cetuximab and panitumumab). The risk of hypomagnesemia is related to the length of treatment and in general is reversible [18]. A meta-analysis estimates that the world incidence is 17 % [19].

#### Clinical manifestations

Most patients with hypomagnesemia have no symptoms, as they usually do not appear until the Mg plasma concentration drops below 1.2 mg/dl; furthermore, hypomagnesemia is accompanied by other electrolyte disorders, such as hypopotassemia and hypocalcemia, which makes it difficult to distinguish clinical manifestations due only to Mg deficit. The most frequent symptoms of hypomagnesemia are above all, cardiovascular (nonspecific ECG alterations such as QT interval prolongation, T-wave and U-wave changes among others), and neuromuscular (paresis, tremor, paresthesia, tetany, spasms, nystagmus or convulsions) [20].

#### Treatment

It is not possible to provide recommendations based on tests for administration and dosing of Mg during or after treatment with CDDP or anti-EGFR mAb, but all authors reviewed agree that hypomagnesemia must be corrected (level of evidence IV, grade of recommendation D).

Some authors consider it is appropriate to prophylactically administer Mg routinely from the start of treatment with CDDP during the pre- or post-hydration phase [21]. For replacement, if the patient is asymptomatic or hypomagnesemia is not very severe (Mg >1 mg/dl), the oral route is the first choice, preferably with preparations such as magnesium chloride, magnesium lactate or magnesium oxide. In symptomatic cases or when the Mg concentration is <1 mg/dl, the intravenous route is adequate and the preparation of choice is magnesium sulfate. (level of evidence IV, Grade of recommendation D). (Table 1).

**Table 1:** Empirical recommendations for Mg replacement according to serum levels

1. Correction of Mg deficit:
1a. Severe HypoMg (<1 mEq/l) with life-threatening symptoms:
Administer 16 mEq IV for 1 min of magnesium sulfate.
Then, 4 ml of magnesium sulfate 500 mg (4 mEq/ml) in 250 ml of 0.9 % saline solution or 5 % dextrose
Continue with IV dose as per regime as described in the next section.
1b. Severe HypoMg <1 mEq/l with no life-threatening symptoms:
First day: 1 mEq/kg and then 0.5 mEq/kg/day for 2–5 days until correction.
Administer magnesium sulfate 500 mg (4 mEq/ml) in 250 ml of 0.9 % saline solution or 5 % dextrose at administration intervals of 1.5 h and speed ≤1.2 mEq/min. Increase infusion time to 4 h if undesirable effects appear such as hypotension.
1c. Hypomagnesemia >1 mEq/l and <1.5 mEq/l without symptoms:
Start with oral route; diarrhea is a limiting adverse effect, but infrequent at a dose of <80 mEq/24 h and also reduced if dose is increased gradually and if Mg is administered after meals. No oral form is indicated for replacement of Mg and it is usually used as antacids or laxatives.
Magnesium oxide tablets: start with 400 mg twice a day with meals and increase dose every week by 400 mg/day. Maximum dose: 400 mg/4 times a day (80 mEq/24 h); there is also oral magnesium chloride and magnesium lactate. If using magnesium oxide oral suspension: 5 ml/4 times a day with meals (56 mEq/24 h).
Consider 6–12 months of treatment to replace Mg deficit.
2. Maintenance for oral correction.
Used to prevent recurrence of deficit. Oral Mg is prescribed in doses of approximately 48 mEq/24 h, divided into various intakes with meals.
Adapted from Crosby et al. [16].

Mg plasma levels should be monitored; an overdose could cause oliguria, diminished level of consciousness and areflexia. Concomitant hypocalcemia and hypopotassemia should also be corrected. Patients with renal failure should receive 50 % of the usual Mg dose if serum creatinine is higher than 2.

### Hypermagnesemia [22, 23]

Hypermagnesemia is any increase in the value of plasma Mg above 2 mg/dl. It is rare in clinical practice, even in patients with renal failure. It is considered mild if between 2.3 and 3.6 mg/dl, moderate between 3.6 and 4.8 mg/dl, serious between 4.8 and 7.2 mg/dl, severe between 7.2 and 12 mg/dl and very severe above 12 mg/dl.

The main causes are chronic renal failure and some cases of acute renal failure, Mg overdose (intoxication), adrenal insufficiency, and hypercatabolic states and tumor lysis syndrome (TLS).

#### Clinical manifestations

It is usually asymptomatic until serum Mg exceeds 4 mg/dl. Between 5 and 8 mg/dl, it may cause nausea and vomiting, skin rash, bradycardia and hypotension. If more severe, it can lead to the elimination of osteotendinous reflexes and somnolence. Above 15 mg/dl, it causes respiratory depression and muscular paralysis, and over 20 mg/dl can cause cardiac arrest with asystolia.

#### Treatment

If asymptomatic, the recommendation is to suspend the administration of Mg. If intense and symptomatic, dialysis is indicated and cardiorespiratory support may be necessary. If the clinical situation is severe, treatment may be initiated with IV calcium gluconate as Mg antagonist (100–200 mg in 5–10 min) until the start of dialysis.

## Potassium disorders

### Introduction

#### Potassium homeostasis

Potassium imbalances are potentially life –threatening; therefore, values found outside of the normal range will typically require immediate therapeutic action and a comprehensive search for an underlying cause. More clinical conditions are associated with aberrant potassium metabolism, and patients with cancer are at particular risk for both those found in the general population as well as those that are tumor or treatment related. Because of the multiple factors involved, the following guideline encompasses our general practices for potassium homeostasis. Its evidence comes from documents or opinions of committees of experts and/or clinical experiences of authorities of prestige; so all the recommendations about potassium disorders have level of evidence IV and grade of recommendation D.

Potassium is the most abundant intracellular cation in the body, with approximately 98 % of the body’s potassium found intracellularly and only 2 % present extracellularly. In Fig. 1, the regulators of potassium distribution between intracellular and extracellular compartment appears. Potassium plays an important role in many biochemical processes, and in the excitatory properties of the membranes of muscle and nerve cells. Normal serum potassium levels are between 3.6 and 5.0 mmol/L. The kidney is the major route of potassium excretion, accounting for 90 % of potassium loss daily. The remaining 10 % is excreted through the gastrointestinal tract. The kidney is, therefore, responsible of the long-term potassium homoeostasis, as well as maintenance of serum potassium concentration [24–26].

#### Workup of potassium imbalances

It is necessary to evaluate the adequacy of the renal response to potassium; it is therefore important that urinary potassium to serum potassium ratio be corrected for urinary concentration. This ratio is referred to as transtubular potassium gradient or TTKG. TTGK >5 in hyperkalemia and <1 in hypokalemia. The cutoff values of >5 or <1 are indicative of non-renal cause for K+ disturbances. This formula can canot be used if urine is more diluted than the serum or contains very little sodium. The next step is to know the urinary potassium excretion:

$$ {\text{TTKG = }}\frac{{{\text{Urinary}}\,{\text{K}}^{ + } / {\text{Serum}}\,{\text{K}}^{ + } }}{{{\text{Urinary}}\,{\text{osmolality/Serum}}\,{\text{osmolality}}}} . $$

### Hyperkalemia

Hyperkalemia is defined as serum potassium greater than 5.0 mmol/l. The possible causes of hyperkalemia are shown in Fig. 1. Hyperkalemia is often asymptomatic and is discovered on routine laboratory test. Patients with severe hyperkalemia (K + >6.5 mmol/L) may present with generalized weakness, paralysis and cardiac arrhythmia. Generally, the severity of clinical presentation correlates with the severity of hyperkalemia and is reflected in ECG changes. The ECG changes related with the K+ serum level are: Mild and moderate (6, 5 mEq/l K +) cause peaking of the T-wave; severe (≥7 mEq/lK +) is related with prolongation of PR and QRS interval, loss of P-wave and marked widening of QRS; and extreme (≥8 mEq/l K +) can show sine wave and ventricular fibrillation.

#### Management of hyperkalemia (Fig. 2)

In the presence of ECG changes, hyperkalemia should be considered an emergency and treatment should begin immediately with calcium gluconate infusion. This should be followed by use of insulin and glucose to help shift potassium into the cell. The next step is removal of potassium from the body. Table 2 summarizes emergency treatment of severe hyperkalemia.

**Fig. 2:**
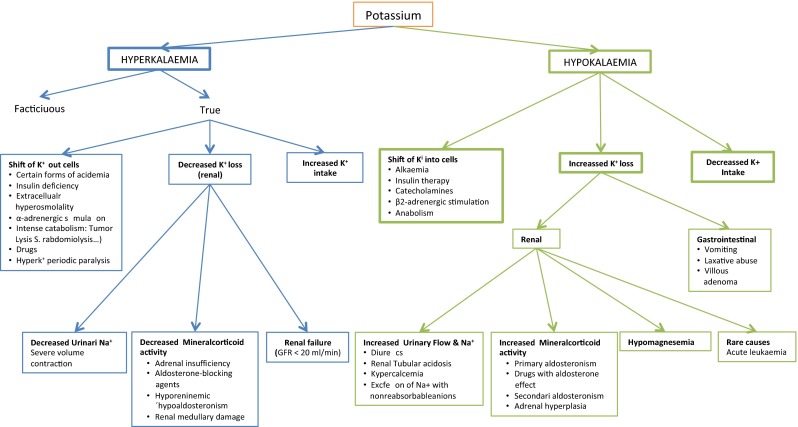
Clinical approach to potassium imbalances (adapted from Bear and Neil [29])

**Table 2:** Treatment of hyperkalemia

Mechanism of action	Therapy	Dose and administration	Onset/duration
I. Membrane stabilization (cardiac injury)	Calcium: calcium gluconate 10 %	10 ml iv in 2–5 min	5–10 min/30–60 min
II. Shift of potassium intracellularly	Insulin + glucose	10U regular insulin iv + 50 g of glucose (500 ml 10 % or 50 ml 50 % (Glucosmon^®^) iv	15–30 min/6–8 h
	β2-adrenergic agonist: salbutamol	0.5 mg 100 ml glucose 5 % in 15 min iv 10–20 mg (2–4 ml) nebulized in 10 min	5–8 min/2–3 h
	Sodium bicarbonate (especially in acidemia)	Bicarbonate 1/6 M, 250–500 ml or 50 ml 1 M iv	30–60 min/6–8 h
II. Removal of potassium	Chelating agents: sodium or calcium polystyrene sulfone	Oral:15–50 g/4–6 h (3–6 spoons with water)	1 h/4–6 h
		Enema: 30–100 g/4–6 h (dissolved in 250 ml)	1 h/12 h
	Loop diuretic: furosemide, torasemide	40–200 mg iv 10–100 mg iv with control of renal function	30 min/8 h
	Dialysis	2–3 h	Immediate/duration of dialysis

When hyperkalemia is due to tubular defect in K+ secretion in association with renal failure, we must consider [27]: hydration and volume expansion to increase urine flow rate and sodium delivery to exchange site; use of loop diuretics to increase sodium delivery and stimulate potassium excretion; restriction of dietary potassium intake to nearly 60 mmol/day and use of oral mineralocorticoids such as fludrocortisone in supraphysiological doses.

In cancer patients, it is important to recognize factitious or pseudohyperkalemia as a consequence of marked leucocytosis or thrombocytosis associated with some malignancies or secondary to therapy. Pseudohyperkalemia can be diagnosed when the serum potassium exceeds that obtained from plasma by more than 0.4 mmol/L. It is typically seen when there is marked leucocytosis (≥100 × 109 L−1) or thrombocytosis (≥1.000 × 109 L−1). It has been reported in acute and chronic leukemias and myeloproliferative disorders (polycythemia vera, myelofibrosis) [28, 29].

#### Tumor lysis syndrome (TLS)

TLS is the clinical and laboratory sequelae that result from the rapid release of intracellular contents of dying cancer cells. It is characterized by the release of potassium, phosphorous and nucleic acids from cancer cells into the blood stream, with the potential to cause hyperkalemia; hyperphosphatemia and secondary hypocalcemia; hyperuricemia; and, should usual homeostatic mechanisms fail, death. TLS most commonly follows treatment of hematologic malignancies, such as acute lymphocytic or lymphoblastic leukemia, acute myeloid leukemia and Burkitt lymphoma, but also occurs after treatment of other bulky or rapidly growing tumors, particularly if the patient is highly sensitive to the effects of cytotoxic chemotherapy.

The Cairo–Bishop criteria define this syndrome in the laboratory (uric acid ≥8 mg/dl, potassium ≥6 mEq/dl, phosphorous ≥4, 6 mg/dl and calcium ≤7 mg/dl) and clinically (acute kidney insufficiency, cardiac arrhythmia, seizure, tetany or other symptomatic hypocalcemia). Patients must meet more than two of four laboratory criteria in the same 24-h period within 3 days before to 7 days after chemotherapy initiation. A >25 % increase from “baseline” laboratory values is also acceptable. In the TLS the rapid liberation of potassium into the extracellular fluid will lead to severe hyperkalemia if it exceeds the normal homeostatic uptake of potassium into the liver and muscle cells [29] The therapy should be directed at increasing the clearance of toxic intracellular contents. Prophylactic therapy depends on the risk for TLS given specific patient and disease characteristics. All patients at risk should receive judicious volume repletion and allopurinol before initiation of chemotherapy, with frequent laboratory assessment after the start of chemotherapy.

### Hypokalemia

Hypokalemia (serum potassium level 3.5 mmol/l or lower) is perhaps the commonest electrolyte abnormality confronted by the clinician and also in cancer patients decreased intake, increased translocation into the cells, or, most often, increased losses in the urine, gastrointestinal tract or sweat can lead to a reduction in the serum potassium concentration (Fig. 1). Similar to hyperkalemia, hypokalemia is often asymptomatic, especially in patients with mild hypokalemia (serum potassium 3.0–3.5 mmol/l). Patients with severe hypokalemia (serum potassium of less than 2.5 mmol/l) usually present with generalized weakness and severe hypokalemia can precipitate rhabdomyolysis that manifests as muscle tenderness and swelling. Cardiac arrhythmias are common in hypokalemia. In moderate to severe hypokalemia, changes in ECG are minimal and often limited to the presence of a U-wave [30, 31].

#### Management of hypokalemia (Fig. 2)

The treatment has two objectives: replacement of potassium and potassium loss correction. The treatment depends on the severity of hypokalemia [31]. In mild hypokalemia (K+ 3–3.5 mmol/l), deficiencies can be corrected by foods rich in the element or by supplemental oral potassium. A dosage of 40–100 mmol/d is sufficient for its treatment. For moderate hypokalemia (2–3 mmol/l), oral potassium replacement such as potassium chloride, potassium phosphate or potassium bicarbonate is recommended. In cases of severe hypokalemia (K+ <2.5 mmol/l), potassium is given intravenously, but the rate of potassium administration should not exceed 20 Eq/h (100–150 mEq/day). The concentration of potassium in serum should not exceed 30 mEq per each 500 cc of serum. 20 mmol/day of potassium in oral form per day is generally sufficient to maintain serum potassium concentration within the normal range in patients with increased loss (diuretic treatment) [31].
